# Comparison of outcome of transcatheter aortic valve implantation in patients with advanced age: A systematic review and meta-analysis

**DOI:** 10.1097/MD.0000000000021443

**Published:** 2020-07-31

**Authors:** Shengde Zhu, Han Li, Guoliang Zhang, Shidong Liu, Zijian Li

**Affiliations:** a First Clinical Medical College, Lanzhou University; b Department of Hematology, First Hospital of Lanzhou University, Lanzhou, China.

**Keywords:** aortic stenosis, octogenarian, outcome, transcatheter aortic valve implantation

## Abstract

**Background:**

Transcatheter aortic valve implantation (TAVI) is an effective treatment to aortic stenosis in patients with advanced age. However, age is recognized as one of the most important risk factors. The aim of our study is to compare the outcome of TAVI between octogenarian patients and young patients.

**Methods:**

Randomized controlled trials, cohort studies and propensity score matching studies will be included in our systematic review and meta-analysis to evaluate clinical outcome in octogenarian patients who undergo TAVI. PubMed, EMBASE, MEDLINE, Cochrane Library and Web of Science will be searched using a comprehensive strategy. The related conference proceedings and reference lists of the included studies will also be checked to identify additional studies. Retrieved records, extract information and assess the risk of bias will be screened by two reviewers independently. STATA software will be used to conduct data synthesis. There is no requirement of ethical approval and informed consent.

**Results:**

This study will eventually be published in a peer reviewed journal in the form of a scientific paper.

**Conclusion:**

This study will provide a comprehensive review of the available evidence for the treatment of aortic stenosis in octogenarian patients underwent TAVI. We hope it will provide a relatively comprehensive reference for clinical practice and future relevant clinical trials.

**PROSPERO registration number:**

CRD42020155189.

**Study protocol registry:**

The protocol has been registered in PROSPERO, which is an International Prospective Register of Systematic Reviews. The registration number is CRD42020155189.

**Ethics and dissemination:**

Ethics approval and patient consent are not required as this study is a systematic review and meta-analysis.

## Abstract 2

Supplemental Digital Content is available in the text

## 1 Introduction

With a prevalence that increases rapidly with age in recent years,^[1]^ aortic stenosis (AS) is a serious disease, which shorten lifespan, endanger human health and reduce quality of life. About 5% of people aged >65 years have moderate or severe AS, and patients with untreated severe symptomatic AS have a poor prognosis.^[2,3]^ Besides, the 2-year survival rate for symptomatic severe AS is only 50%, and the annual fatality rate due to delayed treatment can be as high as 10% to 20%.^[4]^

Nearly 30% of severe AS patients with severe symptoms are not eligible for surgical aortic valve replacement (SAVR) because of multiple comorbidities, advanced age, previous surgical history, or high-risk anatomical features.^[5]^ Transcatheter aortic valve implantation (TAVI) has currently become an alternative treatment for severe AS patients who are inoperable or with very high surgical risk.^[6,7]^ In the 2017 guidelines of the American heart association/American college of cardiology (AHA/ACC), it is clearly pointed out TAVI is recommended for AS patients with high-risk surgical operations (stage D), and is a reasonable alternative to SAVR for medium-risk AS patients (stage D).^[8]^

However, age, as an independent risk factor of perioperative mortality and morbidity for TAVI,^[7]^ don’t be discussed in the 2017 guidelines of AHA/ACC.^[9,10]^ The majority of TAVI patients are senile in the United States, with the median age of them being about 80 year^[11]^ and approximately 16% of them being ≥90 years.^[10,12]^ Previous studies compared the outcome of elderly age who was undergoing TAVI,^[13,14]^ but the influence of age for TAVI has no consensus. To further confirm the efficiency and safety of TAVI in octogenarian, we performed this systematic review and meta-analysis to explore the short- to long-term clinical outcomes of TAVI.

## 2 Methods

### 2.1 Protocol registration

The protocol has been registered in PROSPERO, which is an International Prospective Register of Systematic Reviews. The registration number is CRD42020155189 (http://www.crd.york.ac.uk/PROSPERO/).^[15]^ The content of this protocol will follow the preferred reporting items for systematic review and meta-analysis protocols (PRISMA-P) recommendations. We also plan to conduct it in accordance with the Cochrane handbook for the systematic reviews of interventions and preferred reporting items for systematic review and meta-analysis (PRISMA) guidelines.^[16]^

### 2.2 Eligibility criteria

#### 2.2.1 Study types

All randomized controlled trials (RCTs), cohort trails and propensity score matching studies (PSM) that have compared the outcome of TAVI in patients with advanced age will be included without any restrictions of region and language

#### 2.2.2 Patient types

All AS patients aged over 80 years old was diagnosed by echocardiograph, CT and MRI will be included regardless the gender, race, valve style, complication, STS risk score, EuroScore and access site.

#### 2.2.3 Intervention types

In the experimental group, AS patients underwent TAVI whose mean age are over 80 years old. In the control group, patients are younger (mean age <80 years old).

#### 2.2.4 Outcome types

Outcomes were mainly identified by relevant literature and existing clinical practice. The primary outcome is all-cause mortality in hospital, at 30-day, at 1-year and more than 1-year. The secondary outcomes include myocardial infarction (MI), stroke, major or life-threatening bleedings events, major or minor vascular complications, new onset atrial fibrillation, new permanent pacemaker implantation, acute kidney injury (AKI), NYHA III and IV, moderate-severe paravalvular leak, conversion to open heart surgery, device success, left ventricle ejection fraction (LVEF) and intensive care unit length of stay. Besides, all the endpoints reported in the included studies will be collected and evaluated, although we may not mention some of them in this protocol.

### 2.3 Literature searches

The following databases will be searched from the inceptions to present without any language limitations: PubMed, EMBASE, MEDLINE, Cochrane Library, and Web of Science.

All trials assessing the effect of age for patients undergoing TAVI will be fully considered. The combination of the following search terms will be utilized to identify any potential eligible RCTs, cohort studies and PSM studies: “transcatheter aortic valve replacement”, “transcatheter aortic valve implantation” and “aged, 80 and over”. In addition, Congress and conference proceedings will be manually retrieved. Related articles and references of included research will also be tracked to find potential studies. If significant data was incomplete in included study, we will contact the authors to get unpublished data.

### 2.4 Study selection

Two authors (ZSD and LH) will independently select the studies according to the predefined eligibility criteria. Any disagreements regarding the study selection will be solved by a third author (LZJ or LSD) through discussion. The titles and abstracts of all searched records will be read initially. After that, the full-texts of the rest studies will be further assessed if they can meet all eligible inclusion criteria.

### 2.5 Data extraction and management

Two authors (ZSD and LH) will independently perform data extraction by using a standardized data sheet. Any disagreements regarding the data extraction will be solved by a third author (LZJ or LSD) through discussion. The extracted data mainly comprise of title, author, and publication year, details of study, study methods, treatment details, and clinical outcome.

### 2.6 Assessment of evidence and study bias

The quality of included studies will be assessed by grading of recommendations assessment development and evaluation (GRADE), and classified as high quality, moderate quality, low quality, and very low quality.^[17]^

Two reviewers (ZSD and ZGL) will independently assess the included study bias, and any disagreement will be solved by a third reviewer (LZJ or LSD). Cochrane Risk of Bias Tool will be used to assess the potential bias for each included RCTs. It consists of 7 items, and the quality of each item will be evaluated using standard criteria of Cochrane handbook for systematic reviews of interventions.^[18]^ For cohort studies and PSM studies, 9-star Newcastle-Ottawa Scale will be applied, which rates studies based on 8 criteria in 3 sources of bias.^[19]^

### 2.7 Statistical analysis

All analyses were performed using STATA software 15.0 (StataCorp, College Station, TX). Continuous data will be presented as mean difference (MD) or standardized mean difference (SMD) with 95% confidence intervals (CIs), while the dichotomous data will be shown as odds ratios (OR) with 95% CIs. To calculate the summary estimate across all included studies, we will use the random effects model (Dersimonian and Laird).^[20]^ Heterogeneity assessments will be performed using χ^2^-based Q statistics and I^2^ tests. A *P* < .10 or I^2^ > 50% will be considered as significant heterogeneity.

#### 2.7.1 Subgroup analysis

Subgroup analysis will be performed to find more potential information based on pre-set criteria in

(1) different follow-up time,

(2) different type of event and

(3) different types of implanted valve in TAVI patients.

#### 2.7.2 Sensitivity analysis

Sensitivity analysis will also be considered to perform to check the robustness of pooled results by removing low-quality trials.

#### 2.7.3 Assessment of publication bias

The likelihood of publication bias will be assessed graphically through the generation of funnel plots, evaluated using an Egger test. Statistical significance was set at *P* < .05.^[21]^

## 3 Results

The study does not require ethical approval because the meta-analysis is based on published research and the original data are anonymous. And this study will eventually be published in a peer-reviewed journal in the form of a scientific paper.

## 4 Discussion

In our study, we will provide a comprehensive review of the available evidence for the treatment of AS patients with advanced age with TAVI aimed to compare the outcome of TAVI in an octogenarian patient with that in younger patient. We hope the results from our research may provide meaningful evidence for clinical practice and give a valuable reference for future study.

There seem to be some potential limitations for our study. First, we include article without any language limitations, but more article is English, which will result in some biases in pooling data. In addition, according to the initial search result, less random controlled trials and more propensity-match cohort studies will be included in our study, which may have an obstacle to our data pooling and results interpretation. But it will probably help to promote several more reliable conclusions and focus on more precious direction.

## Author contributions

ZSD and LH conceived the idea for this study; ZSD and ZGL designed the meta-analysis; ZGL and LSD provided statistical advice and input; ZSD and LH drafted the protocol; LZJ and LSD reviewed the protocol and provided critical feedback.

## Supplementary Material

Supplemental Digital Content
