# Supplementary material for: Comparison of outcome of transcatheter aortic valve implantation in patients with advanced age: A systematic review and meta-analysis
Source: Medicine (Baltimore). 2020 Jul 31;99(31):e21443. doi: 10.1097/MD.0000000000021443 (PMC7402761; doi:10.1097/MD.0000000000021443)
Supplement: Supplemental Digital Content [file medi-99-e21443-s001.pdf]

Systematic review

To edit the record click *Start an update* below. This will create a new version of the record - the existing version will remain unchanged.

1. * Review title.

Give the working title of the review, for example the one used for obtaining funding. Ideally the title should state succinctly the interventions or exposures being reviewed and the associated health or social problems. Where appropriate, the title should use the PI(E)COS structure to contain information on the Participants, Intervention (or Exposure) and Comparison groups, the Outcomes to be measured and Study designs to be included.

Comparison of Outcome of Transcatheter Aortic Valve Implantation in Patients with Advanced Age: : A Systematic Review and Meta-Analysis

2. Original language title.

For reviews in languages other than English, this field should be used to enter the title in the language of the review. This will be displayed together with the English language title.

3. * Anticipated or actual start date.

Give the date when the systematic review commenced, or is expected to commence.

15/10/2019

4. * Anticipated completion date.

Give the date by which the review is expected to be completed.

15/12/2019

5. * Stage of review at time of this submission.

Indicate the stage of progress of the review by ticking the relevant Started and Completed boxes. Additional information may be added in the free text box provided.

Please note: Reviews that have progressed beyond the point of completing data extraction at the time of initial registration are not eligible for inclusion in PROSPERO. Should evidence of incorrect status and/or completion date being supplied at the time of submission come to light, the content of the PROSPERO record will be removed leaving only the title and named contact details and a statement that inaccuracies in the stage of the review date had been identified.

This field should be updated when any amendments are made to a published record and on completion and publication of the review. If this field was pre-populated from the initial screening questions then you are not able to edit it until the record is published.

The review has not yet started: No

Review stage	Started	Completed
Preliminary searches	Yes	No
Piloting of the study selection process	No	No

Review stage	Started	Completed
Formal screening of search results against eligibility criteria	No	No
Data extraction	No	No
Risk of bias (quality) assessment	No	No
Data analysis	No	No

Provide any other relevant information about the stage of the review here (e.g. Funded proposal, protocol not yet finalised).

6. * Named contact.

The named contact acts as the guarantor for the accuracy of the information presented in the register record.

Shidong Liu

Email salutation (e.g. "Dr Smith" or "Joanne") for correspondence:
Mr Liu

7. * Named contact email.

Give the electronic mail address of the named contact.

1208087969@qq.com

8. Named contact address

PLEASE NOTE this information will be published in the PROSPERO record so please do not enter private information

Give the full postal address for the named contact.

First clinical medical college, Lanzhou University, Lanzhou 730000, Gansu, China

9. Named contact phone number.

Give the telephone number for the named contact, including international dialling code.

18368914891

10. * Organisational affiliation of the review.

Full title of the organisational affiliations for this review and website address if available. This field may be completed as 'None' if the review is not affiliated to any organisation.

Lanzhou university first clinical medical college

Organisation web address:
https://lzdxdyyy.com/

11. * Review team members and their organisational affiliations.

Give the personal details and the organisational affiliations of each member of the review team. Affiliation refers to groups or organisations to which review team members belong. **NOTE: email and country are now mandatory fields for each person.**

Mr Shidong Liu. Lanzhou university first clinical medical college

Miss Hong Xue. Department of Breast surgery, Hospital of Gansu Provincial, Lanzhou, 730000, China

Mr Shuai Dong. Cardiovascular surgery, First Hospital of Lanzhou University, Lanzhou 730000, China

Mr Fuxiang Liang. Cardiovascular surgery, First Hospital of Lanzhou University, Lanzhou 730000, China

Mr Bing Song. Cardiovascular surgery, First Hospital of Lanzhou University, Lanzhou 730000, China

12. * Funding sources/sponsors.

Give details of the individuals, organizations, groups or other legal entities who take responsibility for initiating, managing, sponsoring and/or financing the review. Include any unique identification numbers assigned to the review by the individuals or bodies listed.

Laboratory of Intelligent Medical Engineering of Gansu Province(Grant no. GSXZYZH2018001)

Grant number(s)

13. * Conflicts of interest.

List any conditions that could lead to actual or perceived undue influence on judgements concerning the main topic investigated in the review.

None

14. Collaborators.

Give the name and affiliation of any individuals or organisations who are working on the review but who are not listed as review team members. **NOTE: email and country are now mandatory fields for each person.**

15. * Review question.

State the question(s) to be addressed by the review, clearly and precisely. Review questions may be specific or broad. It may be appropriate to break very broad questions down into a series of related more specific questions. Questions may be framed or refined using PI(E)COS where relevant.

Population: octogenarians with aortic stenosis (AS)

Intervention:Transcatheter Aortic Valve Implantation (TAVI) with no restrictions on the valve style (balloon- or self-expendable valve) or delivery route

Comparison:younger patients with AS (mean age<80 years)

Outcomes: The primary outcome is all-cause mortality in hospital, at 30-day, at 1-year and >1-year. The second outcomes included myocardial infarction (MI), stroke, major or life-threatening bleedings events, major or minor vascular complications, new onset atrial fibrillation , new permanent pacemaker implantation, acute kidney injury (AKI), NYHA III and IV, moderate-severe paravalvular leak, conversion to open heart surgery, device success, LVEF and intensive care unit length of stay.

Style: Randomized controlled trials, cohort studies and propensity score matching studies

16. * Searches.

State the sources that will be searched. Give the search dates, and any restrictions (e.g. language or publication period). Do NOT enter the full search strategy (it may be provided as a link or attachment.)

We will search the databases from the inceptions to present without any language limitations, including PubMed, EMBASE, MEDLINE, Cochrane Library and Web of Science. All trials assessing the effect of age for

patients undergoing TAVI will be fully considered. The combination of the following search terms will be utilized to identify any potential eligible RCTs and cohort studies: transcatheter aortic valve replacement, transcatheter aortic valve implantation and octogenarian, aged, 80 and over. Similar detailed search strategies will also be applied to the other electronic databases. Additionally, we will also perform manual search of the reference lists of the included studies and relevant reviews.

17. URL to search strategy.

Give a link to a published pdf/word document detailing either the search strategy or an example of a search strategy for a specific database if available (including the keywords that will be used in the search strategies), or upload your search strategy.

Do NOT provide links to your search results.

Do not make this file publicly available until the review is complete

18. * Condition or domain being studied.

Give a short description of the disease, condition or healthcare domain being studied. This could include health and wellbeing outcomes.

The incidence of aortic stenosis (AS) increases with age.Nearly 30% of patients with severe aortic stenosis with severe symptoms are not eligible for surgical aortic valve replacement (SAVR) therapy because of multiple comorbidities, advanced age, previous surgical history, or high-risk anatomical features.Transcatheter aortic valve implantation(TAVI) is an effective treatment for patients with severe aortic stenosis who are inoperable or with very high surgical risk, but age is recognized as one of the most important risk factors. The aim of our study was to compare the outcome of TAVI in an octogenarian population with that in a younger population.

19. * Participants/population.

Give summary criteria for the participants or populations being studied by the review. The preferred format includes details of both inclusion and exclusion criteria.

Inclusion Criteria:

All participants aged over 80 years old with aortic stenosis was diagnosed by echocardiograph, CT and MRI will be included regardless the gender, race, valve style, STS risk score, EuroScore and complication.

Exclusion Criteria:

1.patients without aortic stenosis

2.preexisting machanical or bioprosthetic value in any position

20. * Intervention(s), exposure(s).

Give full and clear descriptions or definitions of the nature of the interventions or the exposures to be reviewed.

Transcatheter aortic valve implantation (TAVI) is an effective treatment to aortic valve disease in patients with elderly age.

21. * Comparator(s)/control.

Where relevant, give details of the alternatives against which the main subject/topic of the review will be compared (e.g. another intervention or a non-exposed control group). The preferred format includes details of both inclusion and exclusion criteria.

Younger patients with aortic stenosis underwent TAVI whose mean age are less than 80 years old.

22. * Types of study to be included.

Give details of the types of study (study designs) eligible for inclusion in the review. If there are no restrictions on the types of study design eligible for inclusion, or certain study types are excluded, this should be stated. The preferred format includes details of both inclusion and exclusion criteria.

Randomized controlled trials, cohort studies and propensity score matching studies

23. Context.

Give summary details of the setting and other relevant characteristics which help define the inclusion or exclusion criteria.

24. * Main outcome(s).

Give the pre-specified main (most important) outcomes of the review, including details of how the outcome is defined and measured and when these measurement are made, if these are part of the review inclusion criteria.

All-cause mortality in hospital, at 30-day, at 1-year and >1-year.

* Measures of effect
The Odds Ratios (ORs) of all-cause mortality in hospital, at 30-day, at 1-year and >1-year.

25. * Additional outcome(s).

List the pre-specified additional outcomes of the review, with a similar level of detail to that required for main outcomes. Where there are no additional outcomes please state ‘None’ or ‘Not applicable’ as appropriate to the review

The second outcomes included myocardial infarction (MI), stroke, major or life-threatening bleedings events, major or minor vascular complications, new onset atrial fibrillation , new permanent pacemaker implantation, acute kidney injury (AKI), NYHA III and IV, moderate-severe paravalvular leak, conversion to open heart surgery, device success, LVEF and intensive care unit length of stay.

* Measures of effect
The Odds Ratios (ORs) of myocardial infarction (MI), stroke, major or life-threatening bleedings events, major or minor vascular complications, new onset atrial fibrillation , new permanent pacemaker implantation, acute kidney injury (AKI), NYHA III and IV, moderate-severe paravalvular leak, conversion to open heart surgery, device success.The mean difference(MD) or standardized mean difference(SMD) of LVEF and intensive care unit length of stay.

26. * Data extraction (selection and coding).

Describe how studies will be selected for inclusion. State what data will be extracted or obtained. State how this will be done and recorded.

Two authors will independently perform data extraction by using a standardized data sheet. Any disagreements regarding the data extraction will be solved by a third author through discussion. The extracted data mainly comprise of title, author, and publication year, details of study, study methods, treatment details, and outcome measurements.

27. * Risk of bias (quality) assessment.

Describe the method of assessing risk of bias or quality assessment. State which characteristics of the studies will be assessed and any formal risk of bias tools that will be used.

Cochrane Risk of Bias Tool will be used to assess the methodological quality for each included randomized controlled trials by 2 independent authors. It consists of 7 items, and quality of each item will be evaluated using standard criteria of Cochrane Handbook for Systematic Reviews of Interventions.While for cohort study, we will use Newcastle-Ottawa Scales to evaluate potential bias from three aspects.Any divisions will be settled down by consulting a third author.

28. * Strategy for data synthesis.

Provide details of the planned synthesis including a rationale for the methods selected. This **must not be generic text** but should be **specific to your review** and describe how the proposed analysis will be applied to your data.

Continuous data will be presented as mean difference (MD) or standardized mean difference (SMD) with 95% confidence intervals (CIs), while the dichotomous data will be shown as odds ratios with 95% CIs. To calculate the summary estimate across all included studies, we will use the random effects model (Dersimonian and Laird). Heterogeneity assessments will be performed using $\chi 2$-based Q statistics and I^2 tests. A $P<0.10$ or $I^2>50\%$ will be considered as significant heterogeneity. Subgroup and Heterogeneity analysis will be performed to find more potential information based on pre-set criteria in different follow-up time and different type of event. The likelihood of publication bias will be assessed graphically through the generation of funnel plots, evaluated using an Egger's test. Statistical significance was set at $P<0.05$. All analyses were performed using STATA software 15.0 (StataCorp, College Station, Texas).

29. * Analysis of subgroups or subsets.

State any planned investigation of 'subgroups'. Be clear and specific about which type of study or participant will be included in each group or covariate investigated. State the planned analytic approach.

Subgroup analysis will be performed to find more potential information based on pre-set criteria in (1)different follow-up time, (2)different type of event and (3) different types of implanted valve in TAVI patients

30. * Type and method of review.

Select the type of review and the review method from the lists below. Select the health area(s) of interest for your review.

Type of review

Cost effectiveness	No
Diagnostic	No
Epidemiologic	No
Individual patient data (IPD) meta-analysis	No
Intervention	Yes
Meta-analysis	Yes
Methodology	No
Narrative synthesis	No
Network meta-analysis	No
Pre-clinical	No
Prevention	No
Prognostic	No
Prospective meta-analysis (PMA)	No
Review of reviews	No
Service delivery	No

Synthesis of qualitative studies	No
Systematic review	Yes
Other	No

Health area of the review

Alcohol/substance misuse/abuse	No
Blood and immune system	No
Cancer	No
Cardiovascular	Yes
Care of the elderly	No
Child health	No
Complementary therapies	No
COVID-19	No
Crime and justice	No
Dental	No
Digestive system	No
Ear, nose and throat	No
Education	No
Endocrine and metabolic disorders	No
Eye disorders	No
General interest	No
Genetics	No
Health inequalities/health equity	No
Infections and infestations	No
International development	No
Mental health and behavioural conditions	No
Musculoskeletal	No
Neurological	No

Nursing	No
Obstetrics and gynaecology	No
Oral health	No
Palliative care	No
Perioperative care	No
Physiotherapy	No
Pregnancy and childbirth	No
Public health (including social determinants of health)	No
Rehabilitation	No
Respiratory disorders	No
Service delivery	No
Skin disorders	No
Social care	No
Surgery	No
Tropical Medicine	No
Urological	No
Wounds, injuries and accidents	No
Violence and abuse	No

31. Language.

Select each language individually to add it to the list below, use the bin icon to remove any added in error.

English

There is not an English language summary

32. * Country.

Select the country in which the review is being carried out from the drop down list. For multi-national collaborations select all the countries involved.

China

33. Other registration details.

Give the name of any organisation where the systematic review title or protocol is registered (such as with The Campbell Collaboration, or The Joanna Briggs Institute) together with any unique identification number assigned. (N.B. Registration details for Cochrane protocols will be automatically entered). If extracted data will

be stored and made available through a repository such as the Systematic Review Data Repository (SRDR), details and a link should be included here. If none, leave blank.

34. Reference and/or URL for published protocol.

Give the citation and link for the published protocol, if there is one

No I do not make this file publicly available until the review is complete

35. Dissemination plans.

Give brief details of plans for communicating essential messages from the review to the appropriate audiences.

Do you intend to publish the review on completion?

No

36. Keywords.

Give words or phrases that best describe the review. Separate keywords with a semicolon or new line. Keywords will help users find the review in the Register (the words do not appear in the public record but are included in searches). Be as specific and precise as possible. Avoid acronyms and abbreviations unless these are in wide use.

37. Details of any existing review of the same topic by the same authors.

Give details of earlier versions of the systematic review if an update of an existing review is being registered, including full bibliographic reference if possible.

38. * Current review status.

Review status should be updated when the review is completed and when it is published. For newregistrations the review must be Ongoing.

Review_Ongoing

39. Any additional information.

Provide any other information the review team feel is relevant to the registration of the review.

40. Details of final report/publication(s) or preprints if available.

This field should be left empty until details of the completed review are available OR you have a link to a preprint.
